# No group differences in Traditional Economics Measures of loss aversion and framing effects in bipolar I disorder

**DOI:** 10.1371/journal.pone.0258360

**Published:** 2021-11-09

**Authors:** Zachary Anderson, Kim Fairley, Cynthia M. Villanueva, R. McKell Carter, June Gruber

**Affiliations:** 1 Department of Psychology, Northwestern University, Evanston, Illinois, United States of America; 2 Department of Economics, Leiden University, Leiden, Netherlands; 3 Department of Psychology and Neuroscience, University of Colorado Boulder, Boulder, Colorado, United States of America; 4 Institute of Cognitive Science, University of Colorado Boulder, Boulder, Colorado, United States of America; Shandong University of Science and Technology, CHINA

## Abstract

Bipolar disorder (BD) is associated with impaired decision making, yet few studies have adopted paradigms from behavioral economics to decompose which, if any, aspects of decision making may be impacted. This may be particularly relevant for decision-making processes relevant to known difficulties with emotive dysfunction and corresponding reward dysregulation in BD. Participants with bipolar I disorder (BD; *n* = 44) and non-psychiatric healthy controls (CTL; *n* = 28) completed three well-validated behavioral economics decision making tasks via a remote-based survey, including loss aversion and framing effects, that examined sensitivity to probabilities and potential gains and losses in monetary and non-monetary domains. Consistent with past work, we found evidence of moderate loss aversion and framing effects across all participants. No group differences were found in any of the measures of loss aversion or framing effects. We report no group differences between bipolar and non-psychiatric groups with respect to loss aversion and framing effects using a remote-based survey approach. These results provide a framework future studies to explore similar tasks in clinical populations and suggest the context and degree to which decision making is altered in BD may be rooted in a more complex cognitive mechanism that warrants future research.

## Background

Bipolar Disorder (BD) is a severe psychiatric disorder [1,2] associated with functional disability [3,4], emotion related disturbance [5,6] and high suicide rates [7]. Reward dysregulation is strongly implicated in the onset and persistence of the disorder [8–10]. Given the severe clinical course of BD, a better understanding of processes which contribute to sustained impairment is critical. This preliminary investigation takes a first-step towards documenting decision-making processes in BD using standard behavioral economics paradigms using a feasible remote-based survey approach.

### Reward dysregulation and decision-making in BD

Psychosocial [8,11], neurocomputational [12], and neural [13] models of BD strongly implicate difficulties with reward dysregulation, including heightened anticipation of, and response to, rewards. We highlight several examples. First, adults with BD report greater positive affect in response to potential rewards and positive stimuli [14]. Second, individuals at risk for, or diagnosed with, BD set overly ambitious goals and expend greater efforts to pursue rewards [6,11]. Third, adults with BD report efforts to amplify positive or rewarding mood states [15]. Finally, in adults at risk for BD, increased reward sensitivity has been associated with decreased estimation of the cost of risky decision making [14] and may begin to explain the functional impairment seen in BD. These findings underscore a key relationship between emotion processing and decision making, emphasizes the importance of emotional experience in models of rational choice [16]. However, there are mixed findings when comparing decision making with respect to monetary and non-monetary stimuli [17,18], calling to question the breadth of contexts in which emotion related changes in BD manifest in altered decision making.

Several pieces of evidence highlight potential difficulties in decision making in BD across a variety of contexts. First, diagnostic criteria for mania symptoms feature difficulties with everyday decision making [19]. Second, BD has been associated with poor decision making during risky situations. For example, currently manic BD participants demonstrate increased risk-taking compared to healthy controls on the Iowa Gambling task [20] and the Balloon Analogue Risk Taking task [21]. Third, decision making appears to be impaired with BD participants reporting worse nutrition and exercise habits than healthy controls [22]. Taken together, these findings link emotion dysregulation in BD to decision making, however, previously mentioned mixed findings in the literature [17,18] suggest there is more to the story. Further work using decision-making paradigms uncommonly studied in BD is warranted to unpack this issue.

### Behavioral economics as a window into decision-making in BD

Behavioral economics provides a window through which decision-making processes can be evaluated as deviations from optimal decision making [23,24]. This provides an opportunity to evaluate how BD relates to (non-)optimal economic decision-making. The current work focuses on one important finding in behavioral economics, which is known as the phenomena of preference reversals [25]. In several experiments, it has been demonstrated that participants perceive similar choice options differently due to particular framing. The present project focused on two such framing effects. The first, framing of outcomes, is operationalized through the quantification of loss aversion and an alternative framing scenario. The second, framing of probabilities, is operationalized through another alternative framing scenario. The particular loss aversion task and framing scenarios chosen for this study—explained in more detail below—are very standard, short, and easy to incorporate in an online survey. Therefore, they provide an important benchmark for the evaluation of common behavioral responses.

Loss aversion is defined as the tendency to weigh potential losses more heavily than potential gains [26]. Within non-psychiatric populations, individuals are typically loss averse, requiring a positive amount almost twice as large as a potential loss to offset the negative utility associated with the loss [27]. Some initial studies suggest this concept may be impacted in clinical disorders. For example, several studies to date have examined loss and risk aversion in anxiety disorders [28,29], while additional work links BD to reduced loss aversion [30], decreased loss aversion as a function of increased reward sensitivity [31] and decreased punishment sensitivity [32]. Separately, some initial work has explored risk aversion in depression and BD [33,34] and found deficits in reward processing and decision making during monetary decision-making tasks. While this work marks progress toward uncovering clinically relevant changes in decision making, the present investigation seeks to understand these changes by linking BD diagnosis and its associated mood symptoms to validated behavioral economics tasks while also extending this work into non-monetary domains.

Framing effects in outcomes are a result of reference dependence, in which participants’ likelihood of choosing a risky or safe option depends on whether it is described as a gain or loss. Studies of such framing effects in BD populations have primarily involved presenting gains and losses in a monetary context using tasks taken from the Cambridge Neuropsychological Test Automated Battery [35,36] or monetary variants of the disease task [30] to examine how responses in BD vary when participants are presented with mixed monetary outcomes. Additional work in a healthy population [37] targets framing effects within alternative domains (life vs death or time scenarios) and find domain-specific responses which differ between participants. This work highlights the impact of context on decision making in positively and negatively framed scenarios and underscores the importance of further examining these kinds of frames in BD.

Framing effects in probabilities are a result of probability sensitivity. This sensitivity is highest when probabilities are close to certain (p = 0 or p = 1). This means that a reduction of the probability attached to a certain outcome will have more impact when the outcome was previously certain versus when it had a 50% chance of materialization [38]. Or even more striking, insurance to reduce aversive harms from 1% to 0% will be valued much higher than when reduced from 2% to 1%. This behavioral pattern has also been labeled as the certainty effect. Despite some work documenting the certainty effect in clinically relevant samples [39], we are unaware of other studies that explore this effect in other populations such as BD.

### The present investigation

The present investigation employed validated behavioral economics tasks measuring loss aversion and framing effects using a remote-based survey among adults with a DSM-IV clinically diagnosed history of bipolar I disorder (BD) compared to non-psychiatric healthy controls (HC). We examined both monetary and non-monetary domains (life versus death scenario) to explore how varying contexts influence framing effects in BD. We also extended this work to relate decision making to measures of current mood given previous work that links dysfunctional emotion processing to decision making changes in BD [5]. This provided a unique opportunity to test the following aims:

#### Aim 1: Group differences in loss aversion

We first investigated group differences in loss aversion between our BD and healthy control group. We predicted that the BD group would exhibit decreased loss aversion when compared to the CTL group. Namely, based on substantial evidence in CTL, people focus their attention more on facing a loss than the possibility to acquire a win of similar magnitude, whereas clinical BD patients are likely to fear a loss less. We expected this relationship to manifest at the symptom level as well with more severe current clinical symptoms predicting greater decreases in loss aversion. These predictions are consistent with a general focus on potential positive outcomes in clinical BD patients and was also based on prior literature suggesting that BD may be associated with reduced loss aversion [30,31].

#### Exploratory aim 2: Group differences in framing effects of outcomes

Our second exploratory aim investigated group differences in framing effects related to outcomes. We explored whether the BD group, compared to the CTL group, exhibited altered risk behavior as measured by responses to a binary choice between a gamble and a sure option, under the influence of positively and negatively described frames. To unpack the potential relationship between BD and framing effects, we also explored the effect of clinical symptom severity on this measure and predict that elevated current symptoms will correspond with a greater likelihood that those in the BD group will deviate from normative patterns of behavior. While we are unaware of any previous investigations of non-monetary framing effects in our target population, previous studies have revealed altered decision-making styles in clinical populations for monetary frames [30].

#### Exploratory aim 3: Group differences in framing effects of probabilities

Our third exploratory aim investigated group differences in framing effects related to probabilities. We investigated whether participants diagnosed with BD show a similar response to certainty, as illustrated by probability sensitivity in a framing scenario known as the Allais paradox. Consistent with previous aims, we explored the effect of current clinical symptom severity to further understand the impact of emotion dysregulation present in BD on responses to certainty. We hypothesize that BD is associated with a reduced need for certainty that corresponds with the increased impulsivity that is often associated with this group [17]. We hypothesize that this increase in impulsive behavior will result in reduced sensitivity to probabilities as compared to CTL. Further, we predict that increases in current clinical symptoms will correspond with increased likelihood that participants in the BD group will result in deviations from normative decision making.

## Method

### Participants

Participants were recruited who had previously participated in at least one of three study protocols from the same laboratory at the University of Colorado Boulder or previously at Yale University. Participants were originally recruited using a multi-prong approach which included flyers, online ads, and local outpatient hospitals and mental health centers. They were then contacted by email to determine interest in participating in a brief online survey for the present investigation. Written consent was obtained for all subjects with ethics approval and consent to participate granted by the University of Colorado Boulder (IRB #: 14–0597, 14–0672, 14–0390) or Yale University (IRB #: HSC #0912006070, HIC #1012007722, HIC #1309012679).

Participants included 44 adults diagnosed with BD type I and 28 healthy control (CTL) adults. BD participants were remitted upon entry to the previous studies from which we recruited for this subsequent investigation. However, participants were not restricted to symptomatic status during this follow-up survey. Consistent with previous studies conducted by the same research group [40–42], participants were not excluded on the basis of comorbid Axis I disorders (aside from substance or alcohol abuse/dependence in the past six months) due to the high rate of comorbidity in BD reported in previous studies [43]. Participants in the CTL group had no current or lifetime history of any Axis I disorder upon study entry. Exclusion criteria for all participants upon study entry included head trauma, severe cognitive impairment, stroke, neurological disease, severe medical illness, or current alcohol or substance abuse/dependence in the past six months. Participants were also excluded on the basis of a catch question planted in the online questionnaire. Participants who responded incorrectly to this item (the item asked participants for a specific multiple-choice response) were assumed to be disengaged from the task. See Table 1 for clinical characteristics describing the eligible sample. For additional information about specific medication usage see S1 Table.

**Table 1:** Demographic and clinical characteristics.

Sample characteristics	BD (*n* = 44)	HC (*n* = 28)	Statistic
**Demographic**			
Age (years)	36.18 (12.45)	34.29 (9.72)	*t* = 0.722
Female (%)	63.6% (*n* = 28)	78.6% (*n* = 22)	*χ*^*2*^ = 1.799
White (%)	88.6% (*n* = 39)	89.3% (*n* = 25)	*χ*^*2*^ = 0.007
Education (in years)	16.00 (2.28)	17.41 (2.62)	*t* = -2.332*
Employment (%)			*χ*^*2*^ = 10.293*
Full-time	45.5% (*n* = 20)	75.0% (*n* = 21)	
Part-time	27.3% (*n* = 12)	25.0% (*n* = 7)	
Unemployed (not student)	4.5% (*n* = 2)	0% (*n* = 0)	
Unemployed (student)	15.9% (*n* = 7)	0% (*n* = 0)	
Retired	6.8% (*n* = 3)	0% (*n* = 0)	
Income			*χ*^*2*^ = 2.521
Less than $10,000	22.7% (*n* = 10)	10.7% (*n* = 3)	
$10,000–$25,000	13.6% (*n* = 6)	14.3% (*n* = 4)	
$26,000–$50,000	27.3% (*n* = 12)	28.6% (*n* = 8)	
$51,000–$75,000	18.2% (*n* = 8)	17.9% (*n* = 5)	
$76,000–$100,000	6.8% (*n* = 3)	7.1% (*n* = 2)	
More than $100,000	11.4% (*n* = 5)	21.4% (*n* = 6)	
Number of children	0.41 (0.82)	0.43 (0.84)	*t* = -0.097
Marital status			*χ*^*2*^ = 5.174
Single (not in relationship)	43.2% (*n* = 19)	21.4% (*n* = 6)	
Single (in relationship)	13.6 (*n* = 6)	28.6% (*n* = 8)	
Live-in partner	9.1% (*n* = 4)	14.3% (*n* = 4)	
Married	27.3% (*n* = 12)	32.1% (*n* = 9)	
Divorced	6.8% (*n* = 3)	3.6% (*n* = 1)	
**Clinical**			
Depression Symptoms	17.80 (7.20)	14.25 (2.59)	*t* = 2.977**
Anxiety Symptoms	9.80 (3.96)	8.71 (2.23)	*t* = 2.977
Mania Symptoms	8.34 (2.99)	7.36 (2.93)	*t* = 1.480
#Psychotropic Medications	1.07 (1.06)	0.14 (0.45)	*t* = -4.295**
Age Onset	15.55 (6.47)	--	--
GAF (baseline)	70.39 (10.14)	88.07 (3.86)	*F* = 77.62**

Note:

** p < .01,

* p < .05.

GAF = Global Assessment of Functioning at baseline laboratory visit where clinical intake was conducted. See S1 Table for more detailed medication information.

### Measures of clinical functioning

#### Diagnostic evaluation

The Structured Clinical Interview for the Diagnostic and Statistical Manual of Mental Disorders, Fourth Edition, Text Revision [44] was used to determine diagnostic status at study entry. Trained clinical interviewers (e.g., clinical psychology faculty, doctoral students, post-baccalaureate or advanced research assistants) administered the SCID-IV. See S4 Appendix for additional information about interrater reliability procedures.

#### Current mood symptoms

Participants self-reported current depression and manic symptoms in the past week. Depressive symptoms were measured using the 13-item Beck Depression Inventory-Short Form [45], and self-reported manic symptoms were measured using the 5-item Altman Self-Rating Mania Scale [46]. This allowed us to examine whether variability in mood symptomatology tracked performance on decision-making tasks. Internal consistency for the BDI-SF (α = 0.94) and ASRM (α = 0.66) were acceptable in the present study.

#### Measures of loss aversion

Loss aversion was measured using a validated choice-list procedure from Economics [47]. This task presents participants with a set of binary lottery decisions with all options presented simultaneously and ordered. This implementation is deliberate and is thought to carry a number of advantages [48]: 1. It is efficient. It only requires six choices. 2. This task specifically elicits loss aversion, and not aversion to variance which is conflated with loss aversion in other tasks. 3. Participants are paid truthfully for one of the six choices—selected randomly—thereby reducing the possibility of portfolio effects or second guessing, potentially even reducing demand effects. 4. This specific loss aversion task has external validity as it has successfully predicted behavior outside the experimental context [48].

In practice, the loss aversion task was administered in the following way. Participants faced six decision problems. Each decision problem was a two-option (binary) choice between playing a gamble and a status quo of $0. Each gamble involved two outcomes, a gain of $6 with a probability of 0.5 and a loss of some amount *x* with a probability of 0.5, essentially a coinflip between the two possibilities. The value *x* consisted of a series of values ranging from $2 to $7 and increased in steps of $1 for each subsequent gamble. Participants were presented with all six gambles at once and responded whether they would “Accept” or “Reject” each gamble. For a full list of stimuli see online supplementary materials. The more often the participant refrained from playing the gamble, the more we can infer she disliked the potential loss. This number was the individual measure of loss aversion. Rejecting more than two gambles implies loss aversion as it illustrates a participant’s preference to win an amount higher than the potential loss. In accordance with standard levels of loss aversion—on average people prefer a gain at least twice the size of a loss—which in this task implies a mean of three reject choices, which is also usually found in this task [48] (S1 Appendix). Therefore, in this task, we consider a mean number of rejected choices of 3 as a benchmark against which we can compare the behavior in this study.

#### Framing effects in outcomes

Framing effects in outcomes were measured using a validated set of choice problems [25]. Each participant was presented with four scenarios that were presented serially and were instructed to select one of two options. Within a set, a scenario would either be framed positively (i.e. in terms of how many people could be saved) or negatively (i.e., in terms of how many people would die). Importantly, both sets of scenarios were equal in magnitude to the potential gain or loss presented to subjects, only the frame of the situation differed. Participants read each scenario, one at a time, and were asked to select one of the two options presented to them (S2 Appendix).

#### Framing effects in probabilities

We additionally measured probability sensitivity using a lottery, also known as the Allais paradox. In this setup, participants were presented with two different lotteries, each involving a choice between two options. Each option had a specific outcome and likelihood associated with it. In the first lottery, option A corresponded with a 100% probability of receiving $1 million and option B corresponded with a 10% probability of receiving $5 million, an 89% probability of receiving $1 million, and a 1% probability of receiving $0. In the second lottery, option C corresponded with an 11% probability of receiving $1 million and an 89% probability of receiving $0 while option D corresponded with a 10% probability of receiving $5 million and a 90% probability of receiving $0. Based on previous work, majority of participants will choose option A in the first lottery and option D in the second lottery, which violates expected utility, and can be explained by an increased awareness of the 1% increase from 0% to 1% chance to win nothing in option A (certainty effect), while a similar 1% increase from 89% to 90% to win nothing does not weigh so heavily for participants (S3 Appendix). See S5 Appendix for additional information on compensation for this task.

#### Study procedure

The current study involved four parts. First, participants were recruited for an in-person session. Afterwards, trained interviewers administered the SCID-IV to confirm diagnosis along with other relevant measures at study entry. Second, participants who were recruited for these previous studies were contacted again to participate in an optional online survey questionnaire study. This survey contained all measures that were used in the current work. Third, interested participants were reconsented and completed all survey measures remotely using Qualtrics^™^ including measures of current symptoms, economic decision making tasks, and other questionnaires not relevant to the present investigation. Finally, at the end of the study, participants were debriefed and compensated for their participation.

## Results

### Preliminary analyses

Demographic and clinical characteristics are reported in Table 1. Participants did not differ on any demographic characteristics with the exception of years of education where the CTL group reported a greater number of years of education compared to the BD group and employment where the CTL group was more often full-time employed and less frequently unemployed. As expected, the BD group self-reported greater current symptoms of depression than the CTL group, but groups did not differ on current symptoms of anxiety or mania. As expected, all three symptoms scales were not normally distributed (as verified by the Shapiro-Wilk normality test), a non-parametric Wilcoxon test was performed and verified the same results as the t-tests reported above. Furthermore, self-reported depression and anxiety symptoms were correlated in both the BD (*r* = 0.61, *p* < .000) and CTL (*r* = 0.39, *p* = 0.039) groups. Across all participants, there were no significant gender differences in self-reported current symptoms of depression (*t* = 0.210, *p* = 0.835), anxiety (*t* = 0.938, *p* = 0.354), or mania (*t* = -1.461, *p* = 0.150).

### Aim 1: Group differences in loss aversion

**Fig 1:**
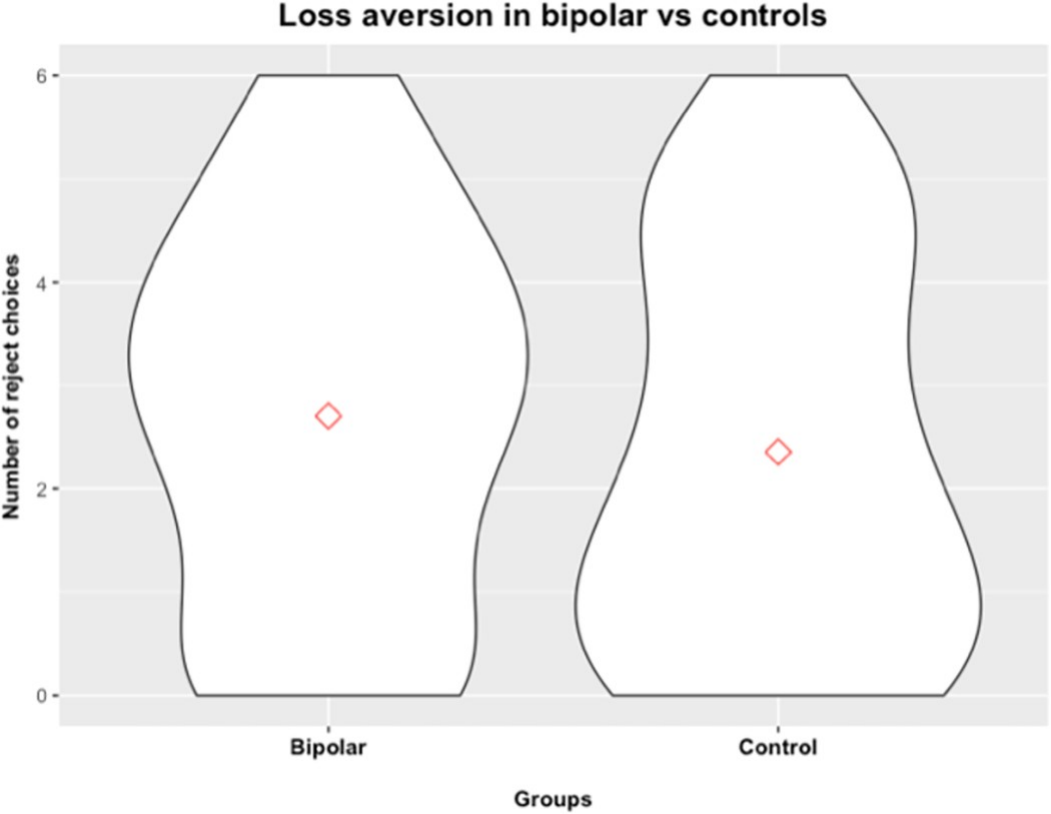
Group comparison in loss aversion. This figure shows violin plots of the scores of loss aversion between our two groups and illustrates the null finding of no difference in loss aversion between BD and CTL.

The first aim examined group differences in loss aversion between the BD and CTL group. Participants across both groups chose to reject the gamble at least some times (*M* = 2.57, *SD* = 1.89 gambles rejected) and were hence moderately loss averse. To examine potential group differences, we performed an independent two-group Mann-Whitney U test and found no differences in loss averse behavior (i.e., number of rejected choices, *W* = 679.5, *p* = 0.461) between the BD (*M* = 2.70, *SD* = 1.86) and CTL (*M* = 2.36, *SD* = 1.95) group. We next regressed the number of reject choices on to ‘group’ (BD served as the reference category) as independent variable while controlling for gender (males served as the reference category), age, education, the interaction of group and gender, and all three current mood scores; the effect of group on loss aversion remained non-significant (*p* = 0.262), further suggesting no difference between groups (Fig 1).

Based on the regression analysis above, it is interesting to note that self-reported anxiety symptoms were associated with increased loss aversion across all participants, indicating that the more anxious a subject felt, the more loss averse they were (*β* = 0.179, *p* = 0.040). Other clinical measures including depression symptoms (*β* = 0.-0.062, *p* = 0.197) and manic symptoms (*β* = 0.037, *p* = 0.650) were not associated with loss aversion. The main results associated with group, as well as secondary results associated with symptom severity, remained qualitatively valid with or without introducing covariates (age, gender, and education) in the model. This was tested by performing a hierarchical linear regression where nuisance covariates were added one by one to test the stability of the estimates related to the variables of interest. Beta estimates for these variables of interest did not vary significantly across these models.

Although not part of our original aim, we note that 14 participants (*BD(N)* = 8, *CTL(N)* = 6) chose to accept all gambles. This behavioral pattern is unusual as it indicates a preference for losses and is a likely indicator of problematic behavior. To understand the effects of these unusual subjects, we conducted two robustness analyses by (1) excluding these participants and (2) adding a dichotomous variable indicating if the participant accepted all gambles (the reference category) or not to our regression model as discussed above. Both these analyses did not change the findings we discussed above qualitatively. After removing these participants however, the median number of reject gambles raised to 3 (reject *M* = 3.19, *SD* = 1.56), which is more in line with past work [48], but there was no significant effect of group on loss aversion (*W* = 439.5, *p* = 0.484).

Finally, we sought to exclude the possibility that this unusual behavior may have correlated with a variable of interest. However, group status (8 loss-seeking BD participants and 6 loss-seeking CTL participants) and gender were matched with the non-loss-preferring sample. This subset of participants also did not score differently on any current mood symptoms.

### Exploratory aim 2: Group differences in framing effects in outcomes

**Fig 2:**
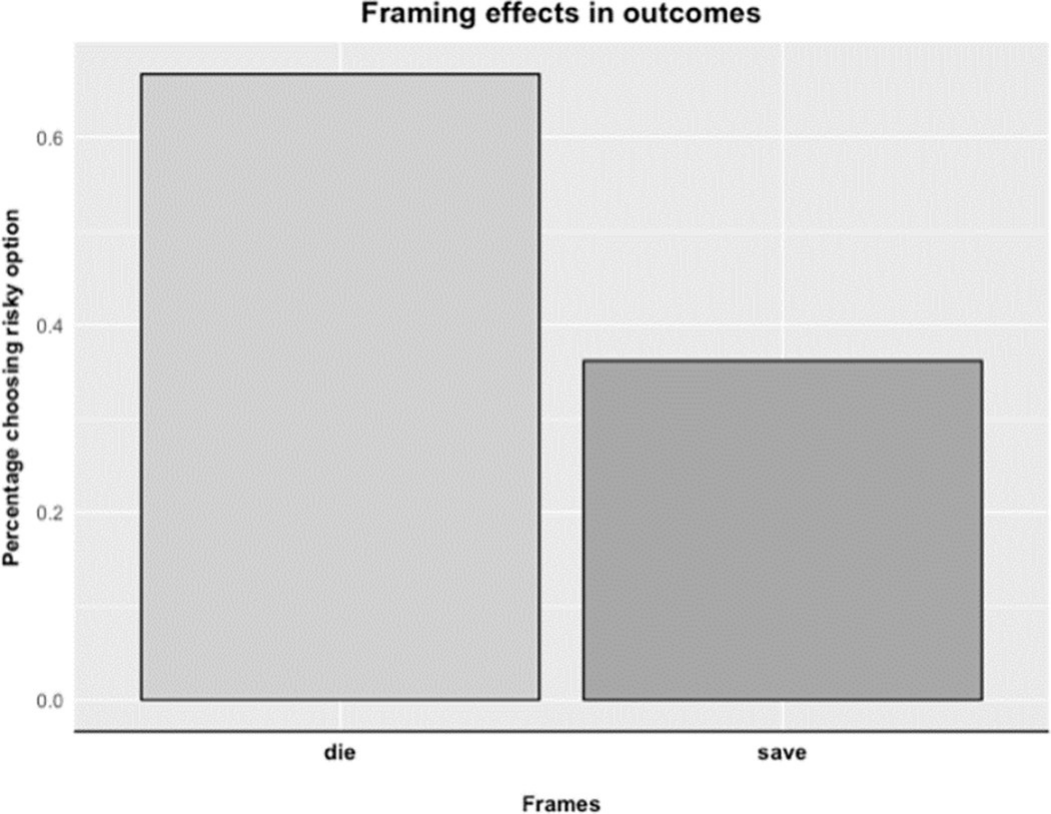
Framing effects across subjects. This figure illustrates the general pattern of preference reversal due to the die/save framing of outcomes in our overall experimental sample. Note that “die” and “save” scenario refer to the relative wordings of framing stimuli (S1b).

The second exploratory aim examined group differences in framing effects between the BD and CTL group. We sought to test whether framing one particular outcome as a loss or a gain (holding how much is lost constant) changes participants’ willingness to choose a risky option. In accordance with standard findings in framing effects, we found that participants across both groups chose the risky option—‘a chance that nobody will die’—more often (χ^2^ = 12.259, p < 0.001, two-sample test for equality of proportions) than the sure option when our decision scenario was framed as a loss (66.67% of all participants) compared to a positive frame (36.11% of all participants, Fig 2). We first performed a two-sample test for equality of proportions on a 2 x 2 table consisting of switching between the risky and sure option for both the positive and negative frame (i.e., did switch vs. did not switch) and group (BD vs. CTL). Results did not reveal significant group differences (χ^2^ = 0.264, *p* = 0.608).

**Fig 3:**
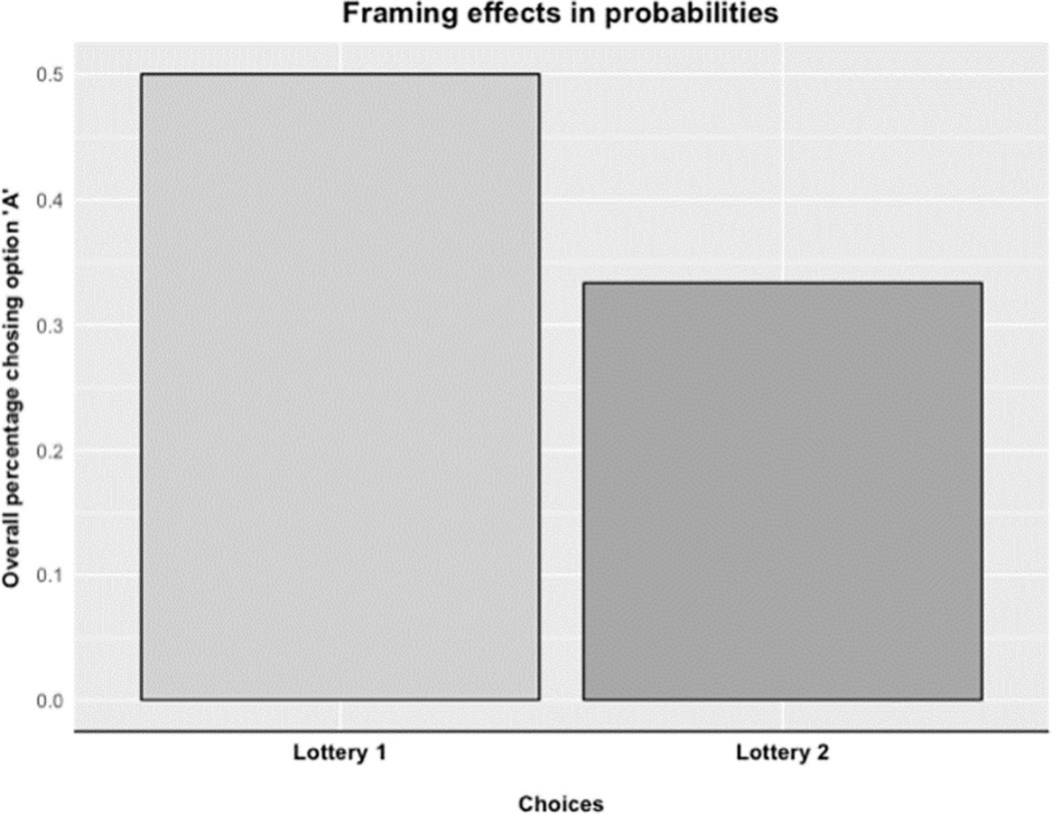
BD by frame interaction effects. This figure illustrates the null effect of a Group x Frame interaction specific to the framing of outcomes in our overall experimental sample. Note that “die” and “save” refer to the relative wordings of framing stimuli (See S1b for study items).

We next ran a logistic regression with the dichotomous choice variable as dependent variable (i.e., risky or sure option). Group (i.e., BD served as the reference category), Frame (i.e., negative frame served as the reference category) and the interaction of Group x Frame, were included as main independent variables. We also controlled for gender (males served as the reference category), age, education and current mood symptom scores. Both the full sample and the analyses with the sample of non-loss seekers illustrated the significance of the main effect of frame (*β* = -1.25, *p* = 0.008), Group (*β* = 1.23, *p* = 0.049) and Gender (*β* = 0.96, *p* = 0.025). In general, the CTL group took more risks than the BD group indicated by the significant coefficient of Group; however, the change in risk preferences as a function of the frame did not differ between CTL and BD as indicated by the non-significance of the interaction between Group x Frame (*β* = 0.52, *p* = 0.514, Fig 3), which was also already indicated by the non-significant results of the two-sample test for equality of proportions. Finally, current mood symptoms were not related to framing effects in outcomes as indicated by non-significant coefficients for interaction terms of symptom scores and frames added to the model above. To test the robustness of this finding, a hierarchical linear regression was performed where nuisance covariates were added one by one to test the stability of the estimates related to the variables of interest. Beta estimates related to the variables of interest did not vary significantly across these models, which demonstrates that results are qualitatively valid with or without the nuisance covariates of age, gender, and education.

### Exploratory aim 3: Group differences in framing effects in probabilities

**Fig 4:**
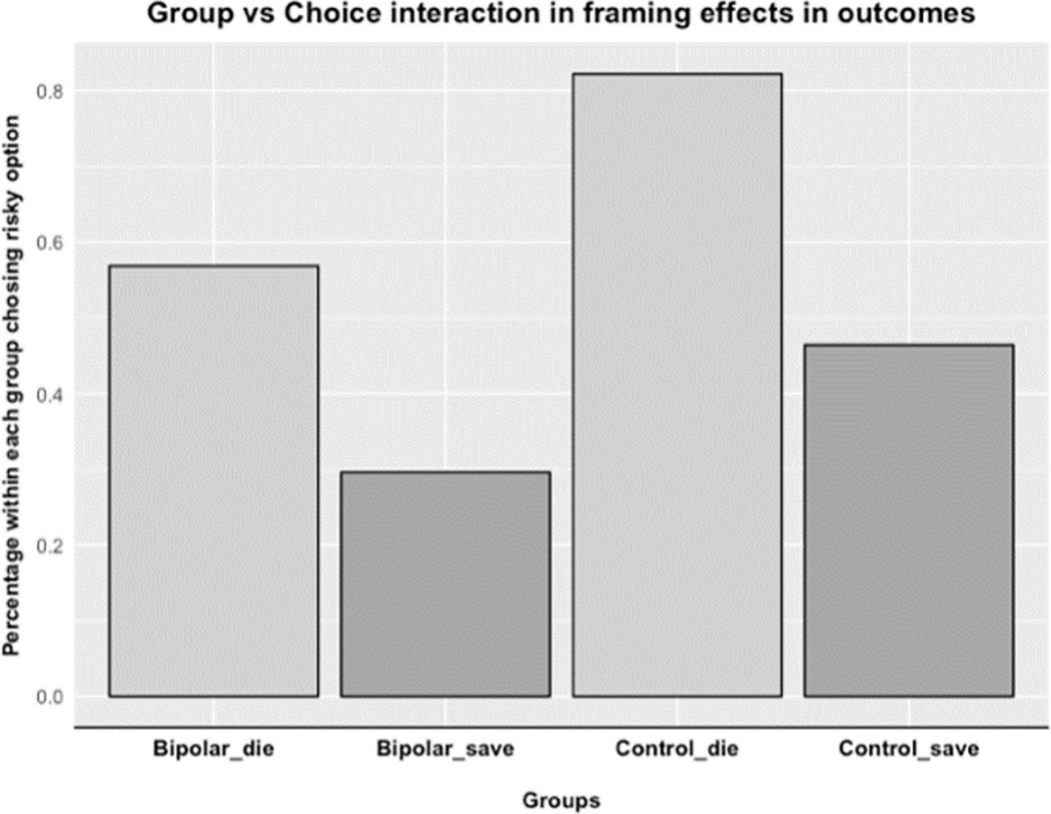
Framing effects across subjects. This figure illustrates standard findings on framing effects specific to probability sensitivity in our overall experimental sample. See S1b for study items.

Our results confirm the typical choice pattern consistent with the certainty effect (option A in the first lottery and option D in the second lottery), as we found that participants try to avoid a decreased sure loss of 1% more often (χ^2^ = 3.457, p = 0.063, two-sample test for equality of proportions) when it decreases from 1% to 0% (50% of all participants) than a decrease from 90% to 89% to receive nothing (33.33% of all participants, see Fig 4), albeit this effect is marginally significant. This choice pattern reflects probability sensitivity, although the effect of this framing effect in probabilities is less strong compared to the framing effect in outcomes. Our analysis did not reveal any difference in the certainty effect between BD and CTL. Namely, a two-sample test for equality of proportions on a 2 x 2 table consisting of switching between option A and option B in both lotteries (i.e., did switch vs. did not switch) and group (BD vs. CTL) did not reveal significant group differences (χ^2^ = 0.018, *p* = 0.893).

**Fig 5:**
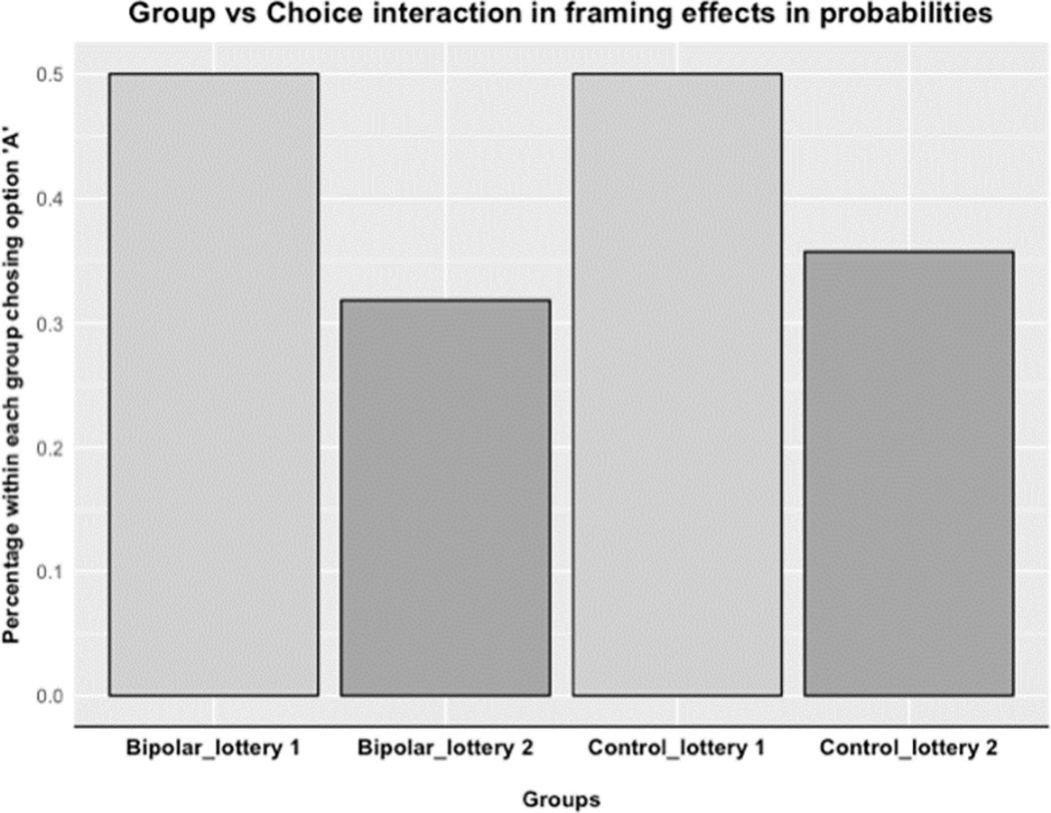
BD by frame interaction effects. This figure illustrates the null effect of a Group x Frame interaction regarding framing effects of probabilities (S1b).

We next ran a logistic regression with the dichotomous choice variable as dependent variable (i.e., option A or B in the respective lotteries) for the full and with the sample of non-loss seekers. Group (i.e., BD served as the reference category), Frame (i.e., lottery with a 1% probability decrease from 1% to 0% served as the reference category) and the interaction of Group x Frame, were included as main independent variables. We also controlled for gender (males served as the reference category), age, education and current mood symptom scores. Both the full sample and the analyses with the sample of non-loss seekers illustrated the marginal significance of the main effect of Frame (*β* = 0.813, *p* = 0.076) and the non-significant main effects for Group (*β* = 0.014, *p* = 0.979) and Gender (*β* = -0.189, *p* = 0.640). The BD and CTL groups did not differ in their sensitivity to probabilities as indicated by the non-significance of the interaction between Group x Frame (*β* = -0.203, *p* = 0.778; Fig 5), which was also indicated by the non-significant results of the two-sample test for equality of proportions. Finally, none of the current mood symptoms related to probability sensitivity after adding interaction terms between symptom scores and Frame to the model above. To test the robustness of this finding, a hierarchical linear regression was performed where nuisance covariates were added one by one to test the stability of the estimates related to the variables of interest. Beta estimates related to the variables of interest did not vary significantly across these models, which demonstrates that results are qualitatively valid with or without the nuisance covariates of age, gender, and education.

## Discussion

The present investigation sought to elucidate disparate patterns of decision making related to loss aversion and framing effects of outcomes and probabilities in bipolar I disorder using novel methods from behavioral economics. Consistent with goals outlined in a recent review highlighting the link between emotion and rational decision making [16], this preliminary investigation was intended to evaluate how effectively measures from behavioral economics might reflect alterations in decision making that may be driven by emotional dysregulation present in BD. Our first aim did not find significant group differences for loss aversion. While several studies report elevated risk-taking behavior in BD populations during a manic episode [20,49], mixed findings are reported in euthymic populations. Moreover, based on the observed association between increased anxiety symptoms and loss aversion, this suggests that altered risk-taking may not be a separate characteristic of BD populations but, instead, may be directly tied to clinically elevated emotions underlying particular episodes associated with BD, which are not present in the current sample. Furthermore, the lack of group differences and our replication of standard patterns of decision making across all tasks supports recent work that has documented the highly episodic nature of BD both with respect to cognitive outcomes [50] as well as neural systems [51]. Current findings may be the result of a return to stable cognitive function within the BD population, which highlights the importance of incorporating such easy to use measures in research and clinical settings where precise measurements of behavior are fundamental to appropriate preventive action. Such findings also emphasize developing models of BD that describe a continuum of clinical experience that may more precisely describe emotive dysfunction and corresponding reward dysregulation in BD [52]. Such dimensions may map more closely to the precise behavioral metrics provided by behavioral economics and future work may wish to explore this idea. It is also possible that the loss aversion task used in this study—an online survey implementation of the task—may not have been sensitive to differences in patterns of decision-making present in BD. More work is needed to uncover the well-described effect of loss aversion in clinical populations and the degree to which a wider range of emotional experiences might differentially impact loss aversion in this population.

Loss aversion describes a pattern of behavior whereby individuals seek to overcome losses by taking more risks that offset losses with subsequent wins [27]. The null effects reported in this study are a departure from much of the literature on risk aversion [53,54], which typically finds deviations from typical patterns of behavior in bipolar populations; however, to date such a distinction is not evident for loss aversion. This is similar to recent findings in anxiety disorders, which reported altered risk aversion when compared to healthy controls, but no differences with regard to loss aversion [28]. Further, it is worth noting there is a significant difference in education between the BD and CTL groups in the current sample. Previous work reports that increases in education appear to correspond with decreases in loss aversion [55]. Given the relatively high degree of education in the CTL group compared with the BD group, group differences in loss aversion may be minimized as both education and the behavioral phenotype of BD counterbalance the effect on loss aversion. Future work should recruit a large sample with an emphasis on education across groups to piece apart this relationship.

The second exploratory aim sought to elucidate how framing effects in outcomes might differ in BD. This was one of the first investigations to test a non-monetary domain under both positive and negative frames in BD. Recent work suggests that clinical symptoms in those previously diagnosed with BD may interact with particular outcome frames in monetary tasks [30,36]. However, the absence of a Group x Frame interaction in our non-monetary domain suggests future work is warranted to explore the extent to which varying degrees of clinical symptoms impact decision making across varying contexts. Future work should strive to capture a broad range of severity with respect to clinical symptoms as the subthreshold BD symptoms present in the current work result in a null effect. Further, given educational differences in risky decision making [55], it’s not clear how the differences in education noted above might influence a Group x Frame interaction in this task. Future work, with larger samples, is needed to piece apart these potential effects.

The third exploratory aim investigated differences between BD and CTL groups in a different kind of framing task. The Allais paradox emphasizes differences in probability sensitivity where small increases in the likelihood of an event (e.g., an increase from 0% to 1%) may have a greater impact on decision making than other shifts in probability (e.g. an increase from 80% to 90%). However, the current study finds the typical pattern of probability sensitivity across both BD and CTL groups. Further, no metrics of current mood appear related to this measure of probability sensitivity. While this aligns with previous work in normative populations [38], it suggests that BD is not more or less sensitive to changes in this domain. Future work may address this question after improving on certain limitations that were present in the current study.

### Limitations and future directions

The current work had several limitations. First, although our sample size is consistent with experimental psychopathology studies in BD, we note that the sample size was modest (*N* = 44 for the BD condition, *N* = 28 for the HC condition). A brief power analysis using the pwr toolbox in the R software suite [56] revealed that, with these sample sizes and a significance level of *α* = 0.05, we have a 90.4% chance of detecting a large effect, a 53.2% chance of detecting a medium effect, and only a 12.9% chance of detecting a small effect. One additional concern with the current findings involves our use of several nuisance covariates, despite the small sample size and correspondingly limited degrees of freedom. However, follow up tests that sequentially introduced nuisance covariates into the model reveal the robustness of all results and suggest analyses related to each of the three target constructs presented in this study remain valid, with or without these additional covariates. Despite this lack of power, it is nonetheless interesting that we validate the standard behavioral pattern in all three tasks in accordance with the wide usage of these tasks and the strong behavioral responses these tasks evoke. This replication of standard patterns of behavior across the BD and CTL samples provides a strong foundation for future work to confidently employ these measures in clinical samples while striving to improve upon the methodological limitations present in the current work. While data from clinical populations are difficult to obtain, the current study can act as a scaffold for future work that may explore these constructs in a sufficiently powered sample.

Second, while we did not exclude participants if they were currently experiencing a mood episode, our measures of current mood symptoms over the past week may not be sensitive to in the moment affective symptomatology that may account for behavioral differences observed in previous studies. Third, it is possible that while we report no significant results with respect to loss aversion and framing effects, additional non-conventional outcomes may provide clinically relevant information. For instance, reaction times during the task may differentiate those with more severe current mania. Previous work that documents increased impulsivity in BD samples supports this hypothesis [54] and could suggest that subthreshold clinical symptoms manifest in subtle reductions in response times across various tasks. While response times were not collected in the current study as it is uncommon to include while assessing these behavioral paradigms, future work might alter the study design to enable these kinds of questions. Fourth, we were only partially successful in attaining a wide symptom profile in our sample. Larger samples are needed to fully explore how mood related symptoms interact with decision making in this context. Fifth, recruitment involved previous studies, some of which finished recruitment up to four years prior to administration of the current study protocol. This time lapse limits our understanding of current clinical diagnoses and potentially relevant clinical experiences. However, this recruitment strategy also provided a unique opportunity to explore how a history of mood related illness alters decision making years after disease onset. Sixth, although remote administration improved the feasibility of the current project, we were not able to monitor study completion in person. It is possible that this may have introduced confounding factors in study administration which may influence study results. Finally, it is possible that the loss-seeking group, who accepted every gamble as part of the loss aversion task, simply did not understand the task properly (although they did pass a catch question included to catch inattentiveness). This creates some uncertainty in the efficacy of an online administration of this task.

The present investigation brings us one step closer to an understanding of altered decision making in BD and raises many suggestions regarding the extent to which these differences exist. In this initial investigation, results support the validity of behavioral economics metrics for use in clinical populations and provides some evidence for the episodic nature of manic symptoms and their impact on economic decision making. Future work should explore cognitive mechanisms that may drive altered decision making in BD, which may involve multifactorial complex interactions. Furthermore, the situations in which these mechanisms become active may be limited to specific mood states or to specific subsets of BD populations which could not be defined in this study.

## Supporting information

S1 Table Psychotropic medication categories. Values refer to frequency of participants in each group taking the medication class. BD = Bipolar disorder group; CTL = Non-Psychiatric Control group.
(DOCX)

S1 Appendix Loss aversion questions. (JPG)

S2 Appendix Framing effects in outcomes. (DOCX)

S3 Appendix Framing effect in probabilities. (DOCX)

S4 Appendix Interrater reliability procedures. (DOCX)

S5 Appendix Compensation procedures for Allais paradox. (DOCX)
