# Supplementary material for: No group differences in Traditional Economics Measures of loss aversion and framing effects in bipolar I disorder
Source: PLoS One. 2021 Nov 9;16(11):e0258360. doi: 10.1371/journal.pone.0258360 (PMC8577741; doi:10.1371/journal.pone.0258360)
Supplement: S1 Table — Values refer to frequency of participants in each group taking the medication class. BD = Bipolar disorder group; CTL = Non-Psychiatric Control group. (DOCX) [file pone.0258360.s001.docx]

BD CTL

| Lithium | 6 | 0 |
| --- | --- | --- |
| Anticonvulsant | 10 | 0 |
| Antidepressant | 8 | 0 |
| Neuroleptic | 7 | 0 |
| Benzodiazepine | 4 | 0 |
| Stimulant | 2 | 1 |
| Sedative-hypnotic | 0 | 0 |
| Other | 1 | 0 |
