# Supplementary figures and images for: No group differences in Traditional Economics Measures of loss aversion and framing effects in bipolar I disorder
Source: PLoS One. 2021 Nov 9;16(11):e0258360. doi: 10.1371/journal.pone.0258360 (PMC8577741; doi:10.1371/journal.pone.0258360)

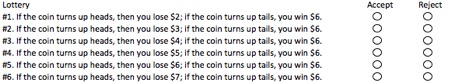

Supplement: S1 Appendix — (JPG) [file pone.0258360.s002.jpg]

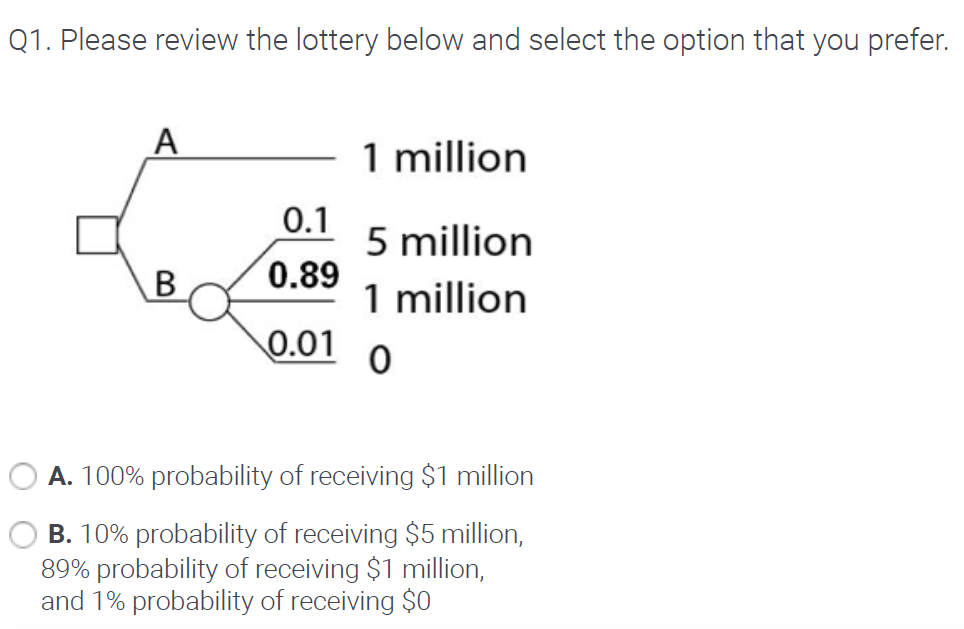


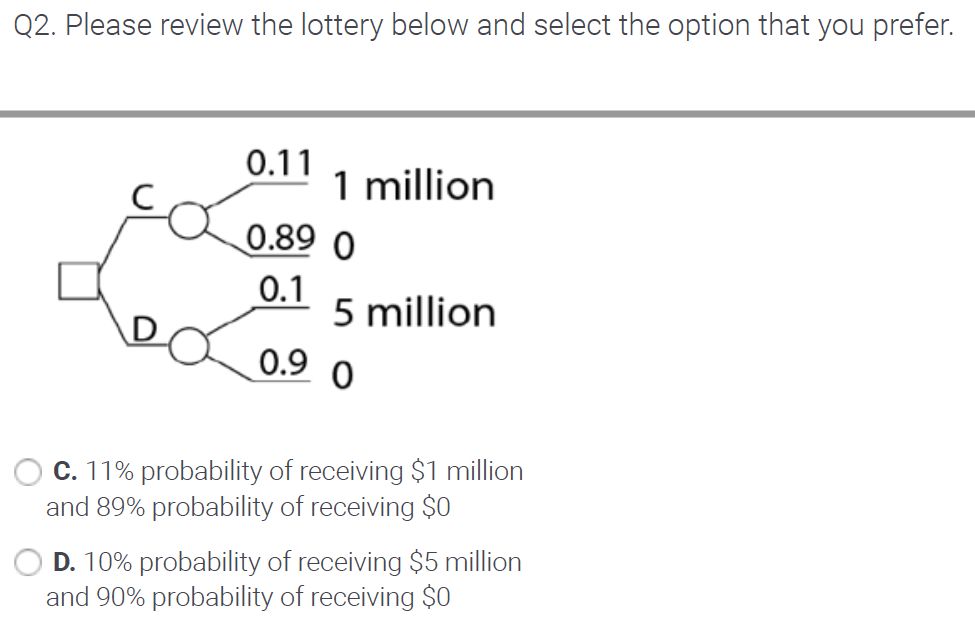

Supplement: S3 Appendix — (DOCX) [file pone.0258360.s004.docx]
