# Supplementary material for: No group differences in Traditional Economics Measures of loss aversion and framing effects in bipolar I disorder
Source: PLoS One. 2021 Nov 9;16(11):e0258360. doi: 10.1371/journal.pone.0258360 (PMC8577741; doi:10.1371/journal.pone.0258360)
Supplement: S2 Appendix — (DOCX) [file pone.0258360.s003.docx]

Imagine the following scenario:

The U.S. is preparing for the outbreak of an unusual Asian disease, that is expected to kill 600 people. Two alternative programs to combat the disease have been proposed. Choose between the following two programs. The exact scientific estimates of the consequences of each program are as follows:

Program A. 200 people will be saved.

Program B. There is a 1/3 chance that 600 people will be saved, and a 2/3 chance that no people will be saved.

Imagine the following scenario:

The U.S. is preparing for the outbreak of an unusual Asian disease, that is expected to kill 600 people. Two alternative programs to combat the disease have been proposed. Choose between the following two programs. The exact scientific estimates of the consequences of each program are as follows:

Program C. 400 people will die

Program D. There is a 1/3 chance that nobody will die, and a 2/3 chance that 600 people will die.
