# Supplementary material for: No group differences in Traditional Economics Measures of loss aversion and framing effects in bipolar I disorder
Source: PLoS One. 2021 Nov 9;16(11):e0258360. doi: 10.1371/journal.pone.0258360 (PMC8577741; doi:10.1371/journal.pone.0258360)
Supplement: S4 Appendix — (DOCX) [file pone.0258360.s005.docx]

We provide additional details on inter-rater reliability (IRR) procedure for interested readers (e.g., Weinstock & Gruber, 2018; Ong, Zaki & Gruber, 2017). IRR for clinical diagnosis and severity ratings was assessed by raters who viewed the interviews and performed initial ratings independently and discussed discrepancies and corrected any errors in scoring that arose as part of discussion during informal consensus meetings with some or all relevant team members present (if a member was not present then that individual consulted with the study PI after the meeting). As such, the IRR ratings reflect corrected (i.e., post-meeting) values. This procedure has been published previously by this research team (e.g., Dutra et al.,2015; Ong, Zaki & Gruber, 2017). Of note, one study (e.g., Hay et al., 2015) from the same lab did not hold informal consensus meetings given a more advanced independent rater performed all the ratings independently (i.e., did not look at the interviewer’s scores beforehand) and so those ratings reported reflect original (i.e., pre-meeting) values. Notably, both interrater reliability approaches from this research group frequently yield strong levels of agreement suggesting good diagnostic accuracy.
