# Supplementary material for: No group differences in Traditional Economics Measures of loss aversion and framing effects in bipolar I disorder
Source: PLoS One. 2021 Nov 9;16(11):e0258360. doi: 10.1371/journal.pone.0258360 (PMC8577741; doi:10.1371/journal.pone.0258360)
Supplement: S5 Appendix — (DOCX) [file pone.0258360.s006.docx]

Compensation consisted of a fixed fee of $5 and a bonus amount of up to $10 dependent on their choices in the Allais paradox and loss aversion tasks. In the Allais paradox, one of the gambles was selected at random and dependent on their chosen lottery, it was played out for real by a random device. The bonus amount for this task was one millionth of the original numbers. In the loss aversion task one of the gambles was selected at random. If the participant decided to reject that chosen gamble, nothing was won or lost. If they had decided to gamble, a random device selected outcomes with a 50% chance of winning $6 or the corresponding losing amount.
